# Case Report: Intraoperative Open-Heart Coronary Angiography in Acute Type A Aortic Dissection

**DOI:** 10.3389/fcvm.2021.731581

**Published:** 2021-09-22

**Authors:** Jue Yang, Xin Li, Zerui Chen, Tucheng Sun, Ruixin Fan, Changjiang Yu

**Affiliations:** Guangdong Provincial Key Laboratory of South China Structural Heart Disease, Department of Cardiac Surgery, Guangdong Cardiovascular Institute, Guangdong Provincial People's Hospital, Guangdong Academy of Medical Sciences, Guangzhou, China

**Keywords:** open-heart, coronary angiography, coronary artery disease, aortic dissection, case report

## Abstract

For patients with acute type A aortic dissection, strongly suspected of having concomitant severe coronary artery disease (CAD), preoperative or intraoperative coronary angiography has been recommended. However, conventional selective coronary angiography in this setting may extend the dissection or aortic rupture. We present the use of intraoperative open-heart coronary angiography in a patient with acute type A aortic dissection. A 50-year-old man presented with chest pain and dyspnea and was admitted to our department with acute type A aortic dissection. The patient underwent coronary artery stent implantation in the left anterior descending coronary artery (LAD) 3 years previously due to an acute myocardial infarction. This time we failed to evaluate the patency of the LAD using multidetector computed tomography. An aortic rupture occurred due to conventional coronary angiography, and open-heart coronary angiography was performed. The examination revealed no significant stenosis. A Bentall procedure and total aortic arch replacement were performed, with an intraoperative stent inserted into the descending aorta, and the patient had an uneventful postoperative course. From this case, we learn that intraoperative open-heart coronary angiography is safe and effective in patients with acute type A aortic dissection.

## Introduction

Acute type A aortic dissection is a life-threatening condition associated with high mortality rates (1). Patients with this disease usually have the same risk factors for coronary artery disease (CAD), and a study has shown that 25% of patients with acute type A aortic dissection have evidence of severe coronary atherosclerosis (2). In cases of acute type A aortic dissection, CAD remains a major predictive factor for both early and late mortality (3, 4) and if possible, it should be identified accurately. Although coronary computed tomography angiography may reveal the conditions of the coronary arteries in most cases, coronary angiography remains the criterion standard in the evaluation of CAD (3, 4). For selected patients with acute type A aortic dissection, strongly suspected of having concomitant severe CAD, preoperative or intraoperative coronary angiography has been recommended by some authors (5–7).

However, conventional selective coronary angiography in this setting may extend the dissection or aortic rupture by advancing the catheter into the false lumen (8). Therefore, a special type of coronary angiography should be performed in these cases. Intraoperative open-heart coronary angiography has been described in a canine model and one patient with aortic valve endocarditis (9, 10). This article will present the use of it in a patient with acute type A aortic dissection and this may be the first time the open-heart coronary angiography was used in this disease.

## Case

**Figure 1:**
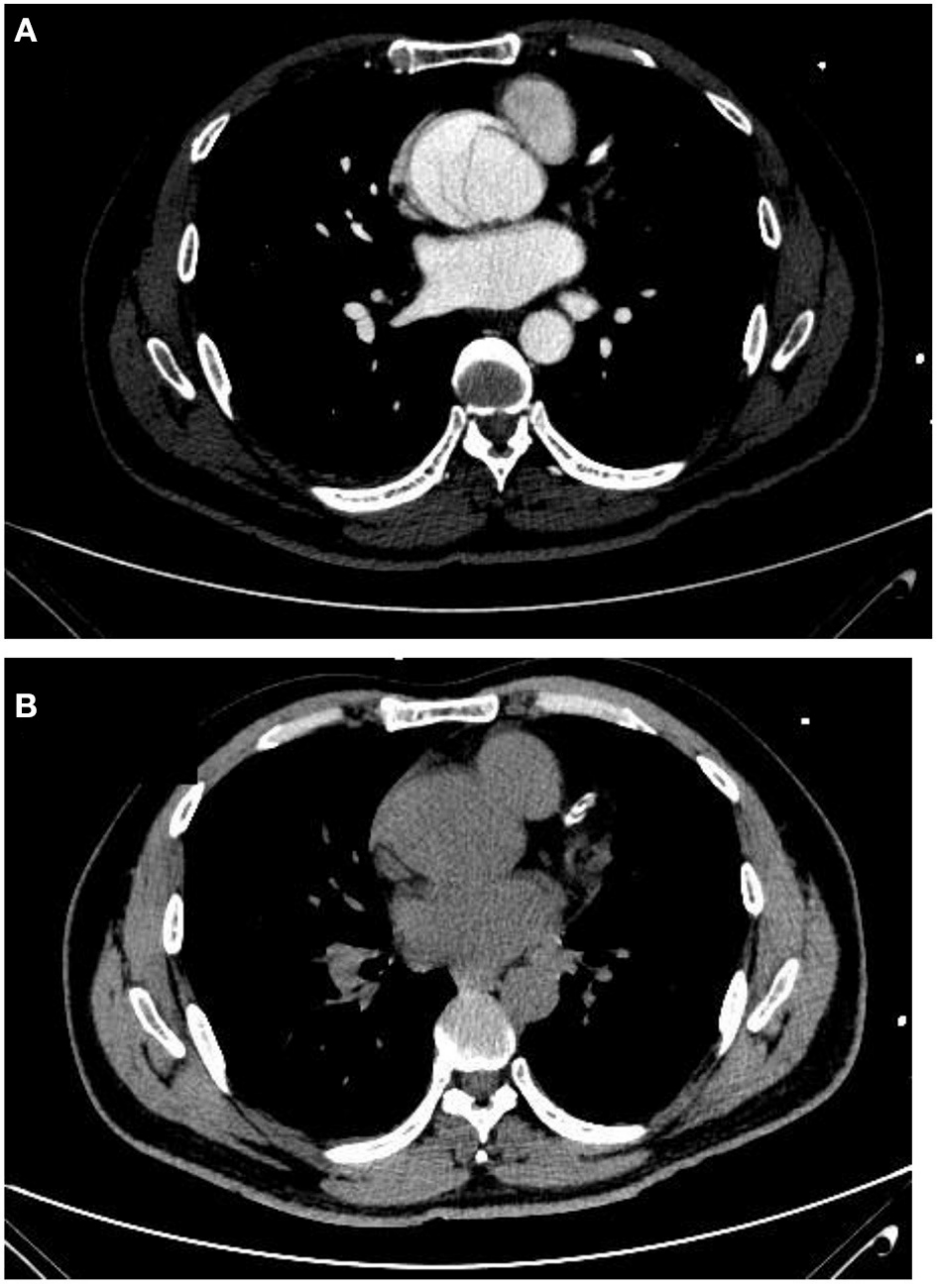
**(A)** MDCT image of the LAD in an enhancement scan. **(B)** MDCT image of the LAD in a plain scan. LAD, left anterior descending coronary artery; MDCT, multidetector computed tomography.

A 50-year-old man presented with chest pain and dyspnea and was admitted to our department with acute type A aortic dissection. The patient underwent coronary artery stent implantation in the left anterior descending coronary artery (LAD) 3 years previously, to treat an acute myocardial infarction. Multidetector computed tomography (MDCT) revealed a severe dissection of the aortic sinus, but evaluating the patency of the LAD was made difficult because of interference from the coronary stent (Figures 1A,B). Therefore, conventional coronary angiography was performed in our hybrid operating room in case of aortic rupture.

**Figure 2:**
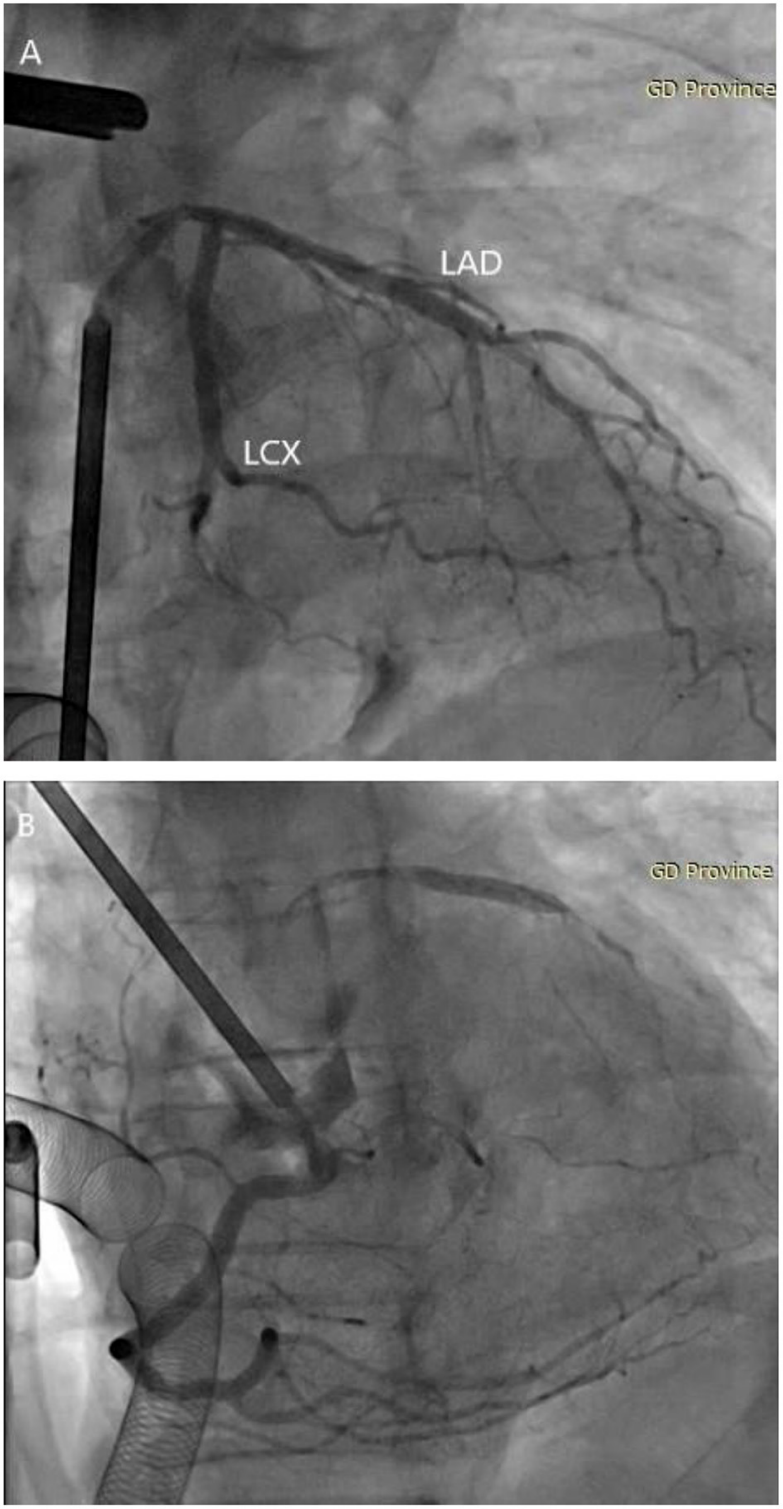
Visualization of the coronary angiography. **(A)** Intraoperative open-heart coronary angiography shows no significant stenosis in the left coronary artery. **(B)** Intraoperative open-heart coronary angiography shows no significant stenosis in the right coronary artery. LAD, left anterior descending coronary artery; LCX, left circumflex coronary artery.

First, we established a cardiopulmonary bypass through the right axillary artery and vena cava. Second, a 5F JL4 angiographic catheter was threaded through the left femoral artery into the ascending aorta. However, an aortic rupture occurred when we tried to select the left coronary artery, therefore an aortic cross-clamp was immediately performed. After perfusion of the cardioplegic solution (1,200 ml cold blood cardioplegia), we performed a coronary angiography by cannulation of both coronary ostia with selective cardioplegia catheters (12 and 14 Ch, Coronary Ostial Perfusion Cannula, Medtronic, Minneapolis, MN, USA). A pump was used to inject 5 milliliters of contrast medium (loperamide, 300 mg iodine/mL, Ultravist, Bayer, Berlin, Germany) at a pressure of 1,000 psi and a flow rate of 4 mL/s, showing no significant coronary artery stenosis in the coronary artery (Figures 2A,B). After injection of the contrast medium, the cardioplegic solution (1,200 ml) was infused through the coronary arteries to prevent contrast toxicity and cardioplegic washout.

Finally, the patient underwent a Bentall procedure and total aortic arch replacement with an intraoperative stent inserted into the descending aorta. The deep hypothermic circulatory arrest time was 20 min. The clamping time was 169 min and the cardiopulmonary bypass time was 292 min. On the first postoperative day, the echocardiography showed the left ventricular ejection fraction was 60%. The patient was discharged safely 14 days later without any complications.

This report was approved by the research ethics committee of Guangdong Provincial People's Hospital and informed consent was obtained from the patient.

## Comment

As a non-invasive examination, coronary computed tomography angiography is the preferred inspection method for evaluating CAD in patients with acute type A aortic dissection. However, coronary computed tomography angiography may be insufficient to quantify significant stenosis in some patients or unfeasible in cases of critical hemodynamic conditions (3, 4). Furthermore, conventional coronary angiography is often a high-risk procedure in patients with acute type A aortic dissection (8). Therefore, we introduced this technique for coronary visualization in patients with acute type A aortic dissection. This technique may prevent the extension of dissection and aortic rupture during preoperative coronary angiography and help determine whether if the patient requires a coronary artery bypass graft. What's more, this open heart technique can be done safely whilst the patient is on cardiopulmonary bypass with little time delayed which differs significantly from other approaches (MDCT and conventional coronary angiography). In our opinion, intraoperative open-heart coronary angiography is safe and effective in patients with acute type A aortic dissection.

## Data Availability Statement

The raw data supporting the conclusions of this article will be made available by the authors, without undue reservation.

## Ethics Statement

The studies involving human participants were reviewed and approved by the Research Ethics Committee of Guangdong Provincial People's Hospital. The patients/participants provided their written informed consent to participate in this study. Written informed consent was obtained from the individual(s) for the publication of any potentially identifiable images or data included in this article.

## Author Contributions

JY and XL wrote the main manuscript text. ZC and TS prepared figures. RF and CY were the managers of the whole study. All authors reviewed the manuscript.

## Funding

This research was supported by the National Key Research and Development Program of China (2017YFC1308003) and the Guangdong Province Science and Technology Department Project (2018YJ025).

## Conflict of Interest

The authors declare that the research was conducted in the absence of any commercial or financial relationships that could be construed as a potential conflict of interest.

## Publisher's Note

All claims expressed in this article are solely those of the authors and do not necessarily represent those of their affiliated organizations, or those of the publisher, the editors and the reviewers. Any product that may be evaluated in this article, or claim that may be made by its manufacturer, is not guaranteed or endorsed by the publisher.
